# Applications of remote epitaxy and van der Waals epitaxy

**DOI:** 10.1186/s40580-023-00369-3

**Published:** 2023-04-30

**Authors:** Ilpyo Roh, Seok Hyeon Goh, Yuan Meng, Justin S. Kim, Sangmoon Han, Zhihao Xu, Han Eol Lee, Yeongin Kim, Sang-Hoon Bae

**Affiliations:** 1 Mechanical Engineering & Materials Science, Washington University in St. Louis, Saint Louis, MO 63105 USA; 2 Division of Advanced Materials Engineering, Jeonbuk National University, Jeonju, 54896 South Korea; 3 The Institution of Materials Science & Engineering, Washington University in St. Louis, Saint Louis, MO 63130 USA; 4 R&D CENTER, M.O.P Co., Ltd, Seoul, 07281 South Korea; 5 Department of Electrical and Computer Engineering, University of Cincinnati, Cincinnati, OH 45221 USA

**Keywords:** Remote epitaxy, Van der Waals epitaxy, 2DLT, Heterogeneous integration, Freestanding membrane

## Abstract

Epitaxy technology produces high-quality material building blocks that underpin various fields of applications. However, fundamental limitations exist for conventional epitaxy, such as the lattice matching constraints that have greatly narrowed down the choices of available epitaxial material combinations. Recent emerging epitaxy techniques such as remote and van der Waals epitaxy have shown exciting perspectives to overcome these limitations and provide freestanding nanomembranes for massive novel applications. Here, we review the mechanism and fundamentals for van der Waals and remote epitaxy to produce freestanding nanomembranes. Key benefits that are exclusive to these two growth strategies are comprehensively summarized. A number of original applications have also been discussed, highlighting the advantages of these freestanding films-based designs. Finally, we discuss the current limitations with possible solutions and potential future directions towards nanomembranes-based advanced heterogeneous integration.

**Graphical Abstract:**

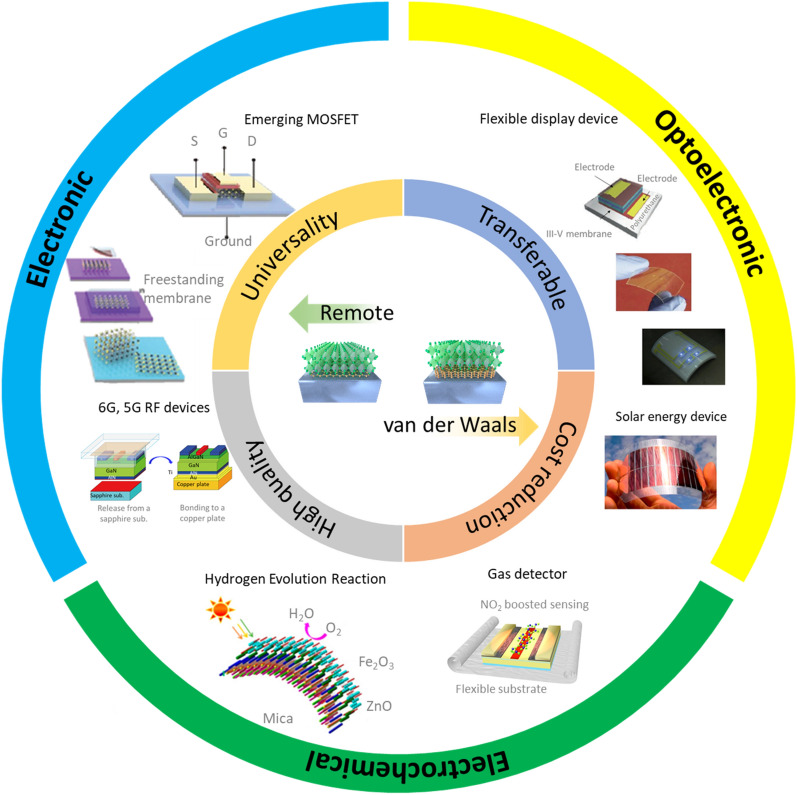

## Introduction

Epitaxy is a material deposition process producing epilayers by copying crystallographic information of substrates. The dangling bond between the crystalline substrate and the atom of the epitaxially formed material induces periodic fluctuations in the surface potential and acts as a driving force for atomic nucleation. Strong chemical bonding is responsible for lattice alignment in epitaxial growth, allowing the epilayer to mimic the crystalline structure of the substrate. The epitaxial layer is thus tightly bonded to the substrate with high binding energy. Due to this strong bonding, it is difficult to separate the epitaxial layer from the growth substrate. However, the demand for detaching the epitaxial layer fields is increasing in various new applications. For example, the separated thin epitaxial layer, called freestanding nanomembranes, can be physically light and flexible because of substantially reduced thickness and stiffness. In addition, it is possible to achieve unprecedented performance and multifunctional function by stacking the ultrathin films of different characteristics and functions that have grown independently on different substrates. These characteristics are vital for novel applications, including flexible-displays, bioelectronics, and the Internet of things (IoT) [1, 2].

To date, several methods such as chemical lift, optical lift, and laser lift-off were developed to separate high quality epitaxial layers from the growth substrates [3, 4]. Chemical lift-off uses a sacrificial layer inserted between the substrate and the epilayer. The sacrificial layer is removed selectively for obtaining a freestanding film. The higher the etching selectivity of the sacrificial layer, the less damage to epitaxial layer is typically observed. Laser lift-off uses the laser energy absorption difference between the epitaxial layer and the substrate. For example, in the GaN and SiC structures, the GaN epitaxial layer can be exfoliated from the substrate because the photon is mostly absorbed in a thin GaN interface layer. Mechanical lift-off is a technology using the cracks near the edge. It can exfoliate epitaxial layer from substrate with crack propagation parallel to the surface. However, these processes include limitations such as few selection of materials, surface damages from breaking strong bond and low throughput of layers [5, 6].

To overcome these limitations, remote and van der Waals (vdW) epitaxy technologies have been proposed as a growth method together with a new layer transfer technique called 2D material-assisted layer transfer (2DLT) [7]. Massive researches have been reported based on this technique. Using 2DLT, the epitaxial layer is grown on a substrate coated with vdW materials. Between the epitaxial layer and vdW materials, there is only a weak physical bond because of no strong covalent bonding in out-of-plane. Thus, the epitaxial layer can be easily separated from weak vdW bonds through the 2DLT.

Here, we review recent progress in advanced epitaxy technologies of remote and vdW epitaxy and their application based on the 2DLT. The unique advantages are discussed that can be obtained from these growth methods, including universality, transferability, cost reduction, and high crystal quality. In addition, representative applications of electronic, optoelectronic, and electrochemical device developed based on these technologies are also explored. Finally, we discuss the current limitations of the two growth methods, as well as possible solutions and future directions towards heterogeneous integration.

## Advanced growth methods

### Remote epitaxy growth

Remote epitaxy is an exemplary material growth method to produce materials on 2D material-coated substrates using remote interaction coming from substrates [8]. The magnitude of the remote interaction is determined by the strength of the polarity of the substrate materials and the vdW gap distance. Therefore, materials having polarity such as III-V, III-N, complex oxide, and halide- perovskite have been explored. It is confirmed that the penetrated potential field of such polar materials gets stronger as their ionicity increases. Also, it is important to have thin 2D materials to utilize remote interaction through 2D materials because thick 2D materials screen potential penetration coming from substrates [8]. Therefore, it is important for successful remote epitaxy to induce uniform field penetration through control of the number of 2D materials [9–11].

Unlike conventional epitaxy, there is vdW bonding at the interface between the grown materials and substrates, allowing exfoliation of the grown materials. This looks analogous to the mechanical exfoliation of spalling, but it is capable of exactly determining the interface where exfoliation happens. As 2D materials have the weakest interface, layer separation occurs at 2D materials. With such feature, precise thickness control of freestanding nanomembranes are achievable through it [12].

### vdW epitaxy growth

VdW epitaxy is the growth of the epitaxial layer on a crystalline substrate, where the epilayer is held together with the substrate by a weak vdW interaction [13, 14]. Since this weak vdW interaction does not have covalent bonding in out-of-plan, the growth layer can be formed a crystallization regardless of the 2D material crystal structure. In addition, since the coated 2D material can screen the electrostatic potential of the growth substrate: it can suppress the strain caused by the lattice constant difference between the substrates and the grown materials, and the epitaxial layer can be grown regardless of whether the substrate is polar or non-polar [15–33]. However, this is accompanied by disadvantages of reducing the quality of grown films by inhibiting the nucleus generation necessary for the formation of crystal on 2D surface. Therefore, to obtain a high quality vdW epilayer, 2D interlayer such as multi-layer graphene should fully screen the electrostatic potential of the substrate is required and finding the optimized growth conditions that can quickly generate nucleus on the surface is also important as well.

In the same way as remote epitaxy, vdW epitaxy adapts a growth structure in which vdW material is inserted between the substrate and the grown materials, freestanding nanomembranes can be obtained with the same transfer method.

## Unprecedented advantages of remote and vdW epitaxy

### Universality

**Fig. 1:**
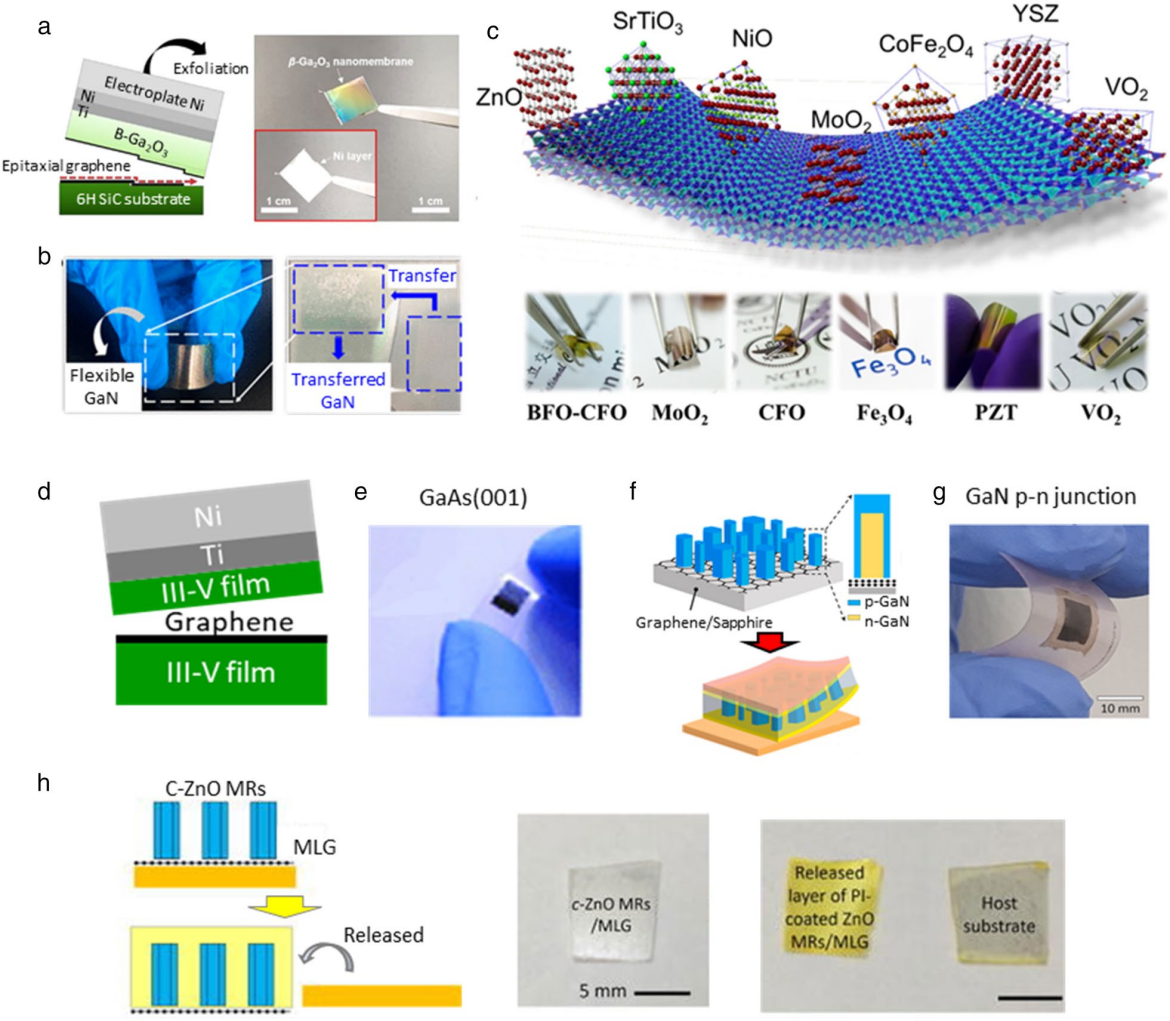
**a**. Schematic illustration explaining the principle of the exfoliation of β-Ga_2_O_3_ from the graphene on SiC and photograph of the exfoliated β-Ga_2_O_3_ nanomembrane; the inset indicates a flip of 180° for the nanomembrane [34], **b**. Photograph image of flexible GaN film transferred from graphene/sapphire substrate [35], **c**. Schematic of various vdW oxide heteroepitaxy of ZnO, MoO_2_, CoFe_2_O_4_, Fe_3_O_4_, PbZrTiO_3_, VO_2_ [22, 36–41], **d**. Schematic views of remote epitaxy of III-V on graphene/III-V substrate and exfoliation of grown III-V layer, **e**. Photographs of single-crystalline GaAs(001) exfoliated from graphene GaAs(001) substrate [42], **f**. Schematic illustration depicting fabrication procedures for flexible GaN-LED array encapsulated with polyimide filler, **g**. Photograph of polyimide encapsulated GaN-LED exfoliated from substrate [43], **h**. Schematics depicting the exfoliation of the heteroepitaxial ZnO microrods process using the thermal release tape technique, and corresponding result sample photographs of ZnO microrods coated by PI [44]

Since remote epitaxy and vdW epitaxy can grow various materials in 2D materials-coated substrates, the limited selection of materials in the conventional growth method can be overcome. The vdW epitaxy growth of β-Ga_2_O_3_ film on graphene/SiC substrates has been reported, where graphene is directly formed on the SiC using high temperature treatment process before growing β-Ga_2_O_3_. The graphene was favorably influenced by lattice arrangements of SiC substate, and thus enabled direct-epitaxy of β-Ga_2_O_3_ on the graphene. Also, the β-Ga_2_O_3_ layer was spontaneously exfoliated at the interface of graphene owing to its low interfacial toughness by controlling the energy release rate through electroplated Ni layers as shown in Fig. 1a [34]. Moreover, Hao et al. grew the high-quality transferable GaN based on the vdW epitaxy method. In this study, they investigated the growth behavior of Al atoms on O_2_-plasma-assisted patterned graphene for improving epitaxial quality. By patterning graphene with O_2_ plasma, the adsorption barrier of Al atoms is greatly reduced and improve the growth quality of the AlN buffer layer. Finally, the high-quality and large-area GaN epitaxial layer with low dislocation density is obtained, and transfer and flexibility are successfully achieved as shown in Fig. 1b [35]. For other materials, various vdW oxide epitaxial layers have been studied. The most common oxide structure has been demonstrated in the heterostructure with epitaxial relationships as shown in Fig. 1c [22, 36–41]. Since there is no strong chemical bonding between the layer and the substrate, the quality of vdW oxide heteroepitaxy depends on the competition of surface energy and the coherency of the symmetry between muscovite and epitaxial layer. Typically, three- or six-fold axis with the lowest surface energy will be the orientation of epitaxial layer on muscovite. In most oxide epitaxial layers on muscovite, the lattice constants and properties were shown to be close to bulk materials or single crystals.

For remote epitaxy, many recent research results have been reported for producing various high quality epitaxial films. Kim et al. recently published research results on the successful growth of III-V (001) epitaxy on III-V (001) substrates using graphene [42]. For example, as shown in Fig. 1(d, e), they demonstrated the feasibility of growing single-crystal GaAs (001) based monolayer graphene overlayers on GaAs (001) substrates and exfoliating grown GaAs (001) from substrate. Furthermore, Hong et al. explained that the remote epitaxy of GaN p-n homojunction microcrystal is demonstrated using metal–organic chemical vapor deposition (MOCVD) for fabricating transferable, flexible while light-emitting diodes as shown in Fig. 1f. Figure 1g shows that the GaN-LED microcrystal peeling off is possible with a weakly bond interface [43]. For other materials, the remote heteroepitaxy of ZnO microrods on the GaN substrate across graphene layer via hydrothermal graphene has demonstrated [44]. Through density-functional theory calculations, they found that charge transfer along the z-direction in multilayer of graphene (MLG)/c-GaN can attract adatoms leading to remote heteroepitaxy. Based on the calculation results, they conducted a remote epitaxy of ZnO microrods experiment using a three-layer MLG, and Fig. 1h shows related results.

### Transferability

**Fig. 2:**
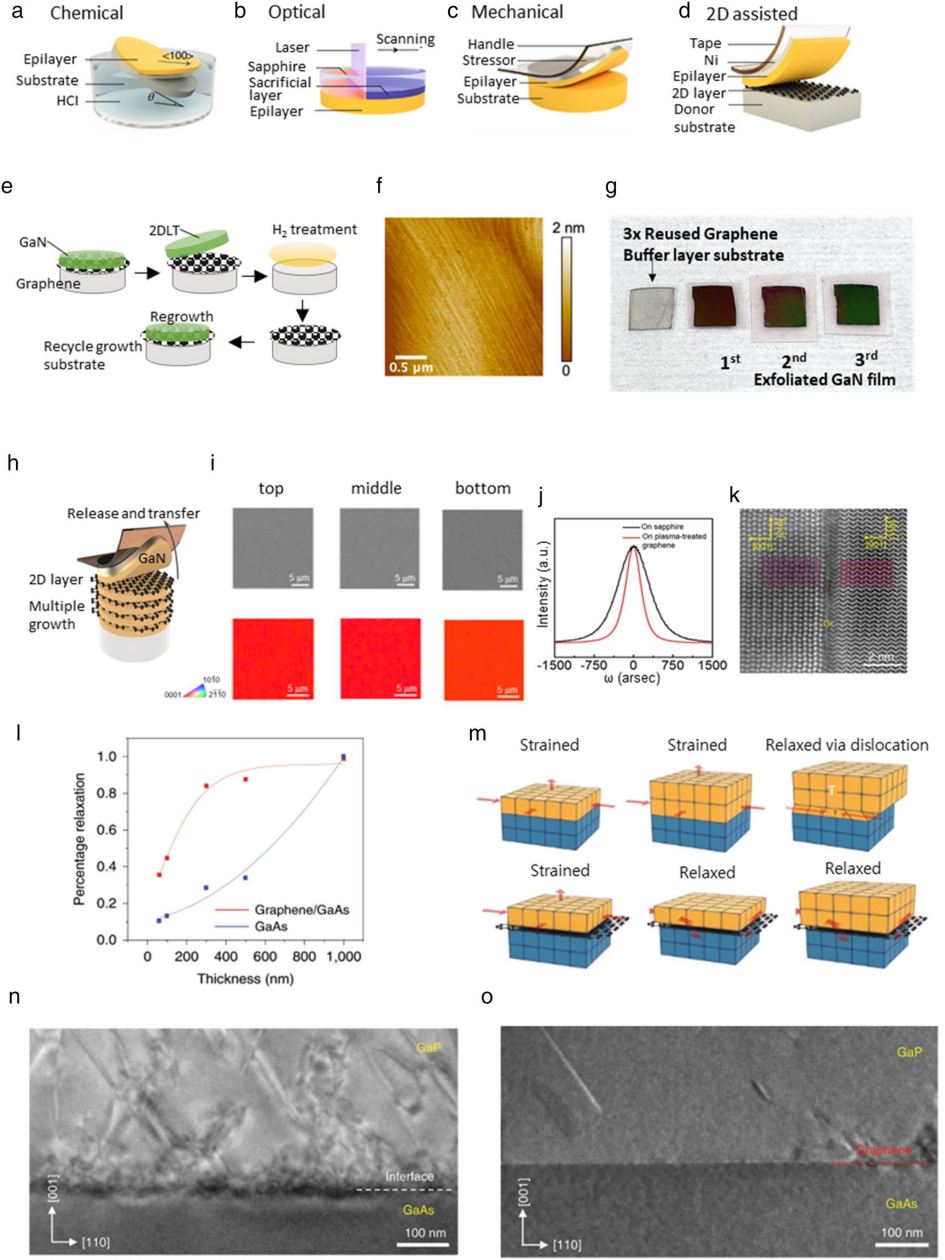
**a**. Schematics of epitaxial lift-off techniques using a chemically etched sacrificial layer, **b**. An optically induced separation between the epilayer and substrate, **c**. Brule-force mechanical spalling using a metal stressor layer, **d**. 2D material assisted layer transfer [64], **e**. Schematic illustration explaining the 2DLT and recycling substrate, **f**. Atomic force microscopy (AFM) image of the surface of the GaN grown on the graphene buffer layer. The RMS roughness is 0.18 nm, **g**. Photograph of the three-times-reused substrate and the exfoliated GaN epilayers [67], h. Schematic of successive multiple membranes production by 2DLT and wafer recycling, **i**. Photographs of exfoliated membrane surface (top) and EBSD map (bottom) [68], **j**. X-ray rocking curves of AlN grown on sapphire (black line) and plasma treated-graphene/sapphire substrates (red line), **k**. Atomically resolved STEM image of the interface of AlN/graphene/sapphire [72], **l**. Strain-relaxation efficiency of InGaP grown on graphene/GaAs and on GaAs [73], **m**. Schematic diagram of strain relaxation via the dislocation, the T indicates where a dislocation forma, and the schematic diagram below is strain via spontaneous relaxation, **n**. Cross-sectional view of GaP grown on bare GaAs, **o**. Cross-sectional view of GaP grown on graphene/GaAs [58]

Since freestanding membranes have a unique vdW interface, they can allow heterogeneous integration of dissimilar materials. This attribute opens an exciting platform for an unprecedented application of electron, photoelectron, and electrochemical with new stacked heterogeneous structure. To implement this, several lift-off technologies have been developed so far. Chemical lift-off is a method of creating freestanding epitaxial films by inserting a sacrificial layer that can be selectively etched in between the epitaxial layer and the substrate as shown in Fig. 2a. For example, for freestanding GaAs, AlGaAs can be etched by hydrofluoric acid whereas InGaAs-based materials are resistant to hydrofluoric acid, allowing AlGaAs to be used as a sacrificial layer [45–51]. Laser lift-off separates epitaxial layers from transparent substrates such as sapphire or SiC using high energy excimer lasers as shown in Fig. 2b. For example, in substrate/GaN structure, a short-wavelength laser is used, which is absorbed by the GaN film, decomposing the substrate/GaN interface into metallic Ga and N_2_ gas, which can then be heated above the melting pint of Ga to separate the epilayer from the substrate [52–54]. Mechanical spalling is a brute force method of creating thin films in micrometers-thick range, where a metal stressor layer is used to initiate a crack running parallel to the substrate as shown in Fig. 2c [55–63]. However, these techniques commonly takes a long time to remove the interlayers of the substrate and film, which limits throughput and harms mass production. In addition, when the epitaxial is mechanically lifted, the roughness of the substrate becomes severe and there is a constraint in the reuse of the substrate.

However, since the two-dimensional layer transfer (2DLT) technology based on vdW and remote epitaxy can easily exfoliate the 2D material between the substrate and the grown nanomembranes as shown in Fig. 2d, the transfer time of the membrane is reduced and the pristine surface remains after exfoliation, [64, 65]. Thus, no wafer refurbishing processes such as chemical and mechanical polishing is required.

### Cost reduction

One significant advantage of the freestanding nanomembranes is their potential to enable wafer recycling [66]. One key stamping block for the widespread application of non-Si semiconductors like GaAs, GaN, SiC, etc. is the expensive material cost arising from the high-cost substrates. However, in most cases the substrate only serves as mechanical support, and only the top thin material layer functions as devices. The freestanding thin films harvested from the remote epitaxy and vdW epitaxy can potentially enable wafer recycling after the exfoliation of the top functional material layers from the 2D material (generally graphene)-coated substrates as shown in Fig. 2e [67]. The atomic force microscopy (AFM) image in Fig. 2f shows a smooth as a grown surface. Figure 2g shows that 2D based growth technique enables reusing of the same substrate three times without any polishing process. Moreover, in order to improve harvest epitaxial layers, the method of growing multiple epitaxial membranes method has been researched as shown in Fig. 2h [68]. In this method, 2D materials are directly formed on substrates in epitaxy systems, which enables an advanced remote epitaxy scheme comprised of multiple alternating layers of 2D materials and epitaxial layers that can be formed by a single epitaxy run. Figure 2i shows the measurement data of the exfoliated surface by the 2DLT process after multi-epithelial growth. Through this result, it was confirmed that the GaN epitaxial layer has a smooth surface shape without any damage during the exfoliation of upper layer.

### High crystal quality

Applying an ultra-thin atomic layer between the epitaxial layer and substrate in the heteroepitaxy system is an important technology that can grow high crystal quality films without dislocation in not only lattice matched but also lattice mismatched conditions. In the vdW epitaxy method, the single-crystal thin films can be grown by effectively reducing the mismatch effect due to the weak bonding between III-N and graphene and alleviating the self-heating problem [12, 14, 68–71]. Liu et al. successfully grew single-crystal AlN by improving III-N nucleation on graphene using plasma-treated graphene [72]. In this research, through DFT calculations, it was found that pyrrolic nitrogen in graphene introduced by plasma treatment facilitates AlN nucleation and enables fast growth. Figure 2j shows that the (0002) full width at half maximum (FWHM) of the X-ray ω-scan (rocking curve) of the AlN epilayer is significantly reduced from 698.6 to 315.5 arcsec with the assistance of graphene. Also, Fig. 2k shows STEM images of AlN/plasma treated graphene/sapphire grown by vdW based on calculated results. The results show that single crystals of AlN were grown on sapphire substrates without dislocations.

In the remote epitaxy growth method, the research about the mechanism of relaxing misfit strain in heteroepitaxial films that can enable effective lattice engineering was reported [73]. Figure 2l shows the InGaP/bare GaAs and InGaP/Graphene/GaAs with 0.74% tensile strain for demonstrating graphene-based strain relaxation. In these structures, the result shows that the relaxation gradually increases as the thickness of In_0.4_Ga_0.6_P increases, and In_0.4_Ga_0.6_P layer grown in graphene/GaAs is particularly spontaneously relaxed. Furthermore, this result means that as assumed in Fig. 2m, there is an alternative relaxation pathway that can be immediately relaxed on the graphene surface. More specifically, if the energy required to displace the bonding at the interface is weaker than that required to introduce a dislocation, the strain could be relaxed without introducing a dislocation. In order to explain the theory more clearly, an experiment was conducted. Figure 2n, o show two TEM cross-section images of GaP grown on GaAs without and with graphene interlayer respectively. The results show that there is a high-density dislocation with significant deformation patterns near the existing heteroepitaxial interface of GaP-GaAs. On the other hand, the dislocation was significantly reduced in the heterogeneous epitaxial interface of the GaP-Gr-GaAs.

## Applications

Freestanding membrane produced by using remote and vdW epitaxy growth methods can be integrated with various other functional membranes. For example, freestanding membrane can be transferred to heat sink substrate, which can improve the heat dissipation property. In addition, the integrated structure with other membranes that change physical properties according to chemical and physical reactions makes it possible to realize the multifunctional devices that can be applied to various areas.

### Electronic application

**Fig. 3:**
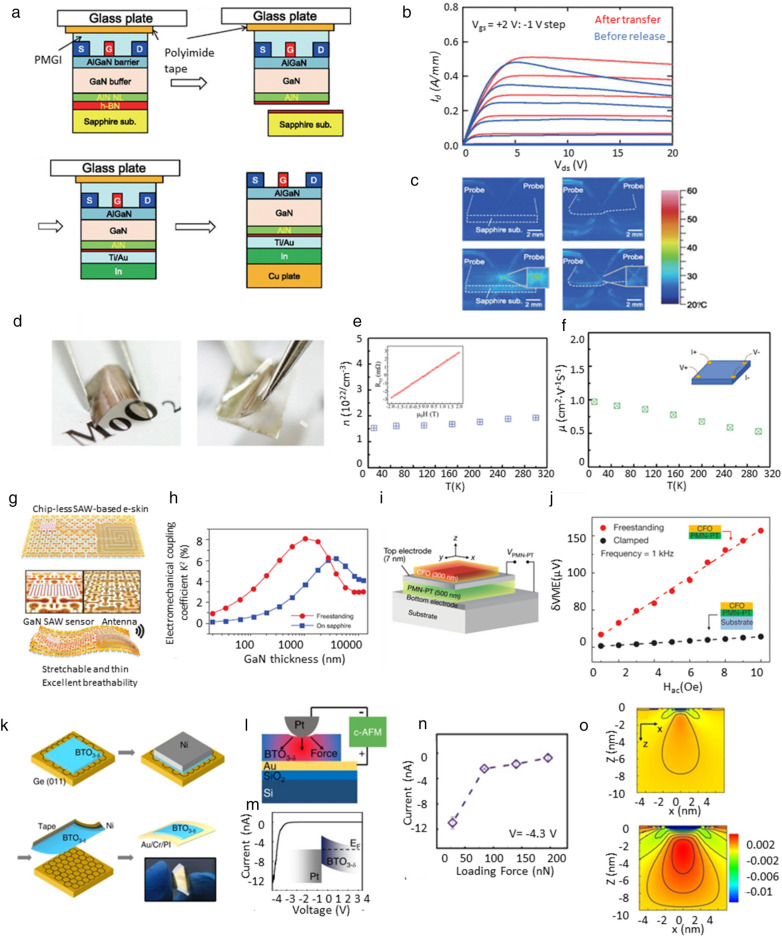
**a**. Schematic views of process sequence for the transfer of an AlGaN/GaN HEMT from a sapphire substrate to a copper plate, **b**. I_d_-V_ds_ characteristics of AlGaN/GaN HEMTs before release from sapphire (blue line) and after transfer to a copper plate (red line), **c**. temperature map of the samples, taken with IR camera Neo Therno 700 (up-left and -right show the off state of the unreleased and transferred devices, respectively, down-left and -right show the on state oof the unreleased and transferred devices, respectively) [15], **d**. the flexibility of MoO_2_/muscovite sample(left) and removal of MoO_2_ film from muscovite substrate, **e**. carrier concentration of MoO_2_ films do not show any obvious temperature dependence, **f**. mobility in the MoO_2_ films do not show any obvious temperature dependence [22], **g**. Chip-less wireless e-skin based on surface acoustic wave (SAW) devices made of GaN freestanding membranes, **h**. Calculated electromechanical coupling coefficient (K^2^) of GaN SAW devices by the function of GaN thickness [74], **i**. The device structure of CFO/PMN-PT magnetoelectric coupling device. In the case of clamped device, the substrate and the bottom electrode are STO and SRO, respectively. In the case of the freestanding device, the substrate and the bottom electrode are PDMS and Ti, respectively, **j**. The voltage generated across the PMN-PT film in response to the magnetic field input [75], **k**. Schematic illustration of the BTO film growth, exfoliation, and transfer process onto a flexible PI substrate, **l**. Illustration of the experimental configuration of the c-AFM test, **m**. I-V curve of the Pt/BTO junction, **n**. Evolution of the current as a function of loading force, **o**. COMSOL FEM calculation with a tip-force model of the BTO film under applied force 28nN(up) and 196nN(down). The colors of blue to red correspond to lower to higher strain values [77]

For electronic applications, vdW epitaxy and subsequent transfer can be used to improve heat dissipation required by radiofrequency (RF) devices. AlGaN/GaN high-electron-mobility transistors (HEMT), one kind of RF device, were transferred to a higher thermal conductivity than a growth substrate to suppress the self-heating effect and increase drain currents [15]. As shown in Fig. 3a, an AlGaN/GaN heterostructure was grown on AlN (nucleation layer)/h-BN/sapphire. An AlGaN/GaN HEMT with a Schottky Au/Ni gate electrode and ohmic Au/Ni/Al/Ti source/drain electrodes were fabricated using photolithography and a lift-off process. The AlGaN/GaN HEMT device can be mechanically transferred from the sapphire wafer using the h-BN as a release layer. After the deposition of Ti/Au/In layers on the released AlGaN/GaN HEMT, the HEMT/Ti/Au/In was bonded to a Cu plate using thermal fusion bonding. The AlGaN/GaN HEMT that was not transferred showed a decrease in drain current by 30% with increasing drain voltage above saturation voltage, which is attributed to the self-heating effect. On the other hand, in the transferred AlGaN/GaN HEMT, the decrease in drain current was only 8%, as shown in Fig. 3b. Transfer from the sapphire to the copper plate mitigates self-heating effect because copper has higher thermal conductivity than sapphire in Fig. 3c. Heterogeneous integrated structures with substrates with high heat dissipation efficiency can improve the performance of RF and power devices. The reported electron mobility and sheet electron density of the 2D electron gas at the AlGaN/GaN interface was 1360 cm^2^V^−1^ s^−1^ and 7.4 × 10^12^ cm^−2^.

Flexible electronics are one of the main applications of freestanding epitaxial films prepared by vdW epitaxy. For flexible devices, the conductive bottom layer with a single crystallinity is desirable to epitaxially grow the active layer on top of the bottom electrode. Metallic MoO_2_ with high crystallinity was grown on a flexible muscovite substrate [22]. The similar crystal structures of MoO_2_ and muscovite lead to epitaxial growth even with the weak vdW force between them. The resulting monoclinic MoO_2_ epitaxial layer on the muscovite could be used not only as a bottom electrode but also as a base layer for the heteroepitaxial growth of other oxide layers on top of the MoO_2_ layer. Figure 3d shows the sample flexible MoO_2_/muscovite, which it can be bent without any observable cracks appearing in the system. Due to the weak bonding between the layers of muscovite, it can be cleaved much thinner than the conventional oxide substrates. Furthermore, in order to characterize the charge carrier type and carrier concentration of the MoO_2_ films, temperature dependent Hall measurements were carried out. Figure 3e shows a typical result of the vertical resistance R versus the magnetic field, in the range from − 2 T to 2 T at 10 K. The positive slope indicates that MoO_2_ is p-type. The carrier concentration, n, reaches a value of 1.9 × 10^22^ cm^−3^ and the carrier concentration in this MoO_2_/muscovite system shows almost no temperature dependence. The calculated mobility of MoO_2_ is 0.53 cm^2^V^−1^ s^−1^ at 300 K and 0.97 cm^2^V^−1^ s^−1^ at 2 K, shown in Fig. 3f.

Surface acoustic wave (SAW) devices can be also enabled by piezoelectric films made from remote epitaxy [74]. Single-crystalline piezoelectric GaN film was grown on a 2D material/GaN/sapphire by remote epitaxy. The GaN film was peeled off using a Ni stressor and bonded to the (flexible polyimide film)/(sacrificial metal layer)/(Si wafer) with epoxy adhesives. Subsequently, conventional cleanroom processes were utilized to make a stretchable surface acoustic wave device made of the GaN film integrated with a stretchable antenna in Fig. 3g. A simulation of SAW generation was performed to analyze the performance improvement of the device. The results show that the electromechanical coupling coefficient K^2^ is negligible for substrate-bonded GaN films thinner than 300 nm as shown in Fig. 3h. Substrate binding alters the acoustic mode and substantially reduces the displacement amplitude of acoustic oscillations. However, since the GaN layer in the proposed structure is de-clamped with the substrate, the electromechanical coupling is greatly improved by eliminating the coupling effect. In this SAW based chip-less wireless e-skin, an ultra-thin GaN SAW sensor can cope with chips, circuit parts and sensors. They can also be incorporated into thin patches of perforated polydimethylsiloxane (PDMS) to remove perspiration and skin by-products, providing conformability, long-term wearability and significantly lower power consumption compared to chip-based e-skins.

Moreover, the heterogeneous integration of piezoelectric and magneto-strictive material led to magnetoelectrical coupling mediated by mechanical strain [75, 76]. A Pb(Mg_1/3_Nb_2/3_)O_3_-PbTiO_3_ (PMN-PT)/ SrRuO_3_ (SRO) layers were epitaxially grown on a SrTiO_3_ (STO) substrate. The piezoelectric PMN-PT can be exfoliated from the SRO layer because of the weak bonding between them. Separately, a magneto-strictive CoFe_2_O_4_ (CFO) layer was deposited by remote-epitaxy on a STO substrate. The PMN-PT was transferred to a Ti-coated polydimethylsiloxane (PDMS) substrate. A 7-nm Pt top electrode was deposited onto the PMN-PT layer, and the CFO membranes was transferred to the top of Pt/PMN-PT/Ti/PDMS as shown in Fig. 3i. Then, the device was measured to magnetic induction and electrical coupling effect with the PMN-PT device clamped on the substrate. The stand-alone CFO/PMN-PT device produces a much larger voltage(δVME) than the clamped device (477 mVcm^−1^) with a magnetic cell coupling factor of 27675 mVcm^−1^. These results show that the coupling effect is significantly enhanced when both are freestanding in heterogeneous stature of CFO and PMN-PT as shown in Fig. 3j.

Other than flexible electronics, freestanding epitaxial films can benefit flexoelectric, piezoelectric, and multiferroic devices. Although only materials with non-centrosymmetric crystals show piezoelectric effects, all materials including centrosymmetric materials show flexoelectric properties. In contrast to piezoelectric materials in which uniform strain generate polarization, flexoelectric materials generate polarization in response to strain gradient. The strength of flexoelectric polarization becomes stronger as the flexoelectric film becomes a freestanding film and possesses smaller dimensions because of the increase in strain gradient. As an example, a flexoelectric BaTiO_3_-δ (BTO) film with a cubic phase was remote-epitaxially grown on a graphene-covered Ge substrate [77]. In contrast to tetragonal BTO films, the cubic BTO film did not show piezoelectric property at room temperature, which was confirmed by piezo-response force microscopy (PFM) measurement. The BTO film was exfoliated by using a Ni stressor layer and a thermal release tape and was transferred to a target substrate, such as Au/Cr/polyimide. Figure 3k shows the measurement using conductive-AFM (c-AFM), where the Pt tip of the c-AFM cantilever forms contact with the exfoliated and transferred 10 nm-thick BTO film on the Au/Cr/SiO_2_/Si substrate. Figure 3l exhibits the typical I-V curve obtained from the Pt/BTO/Au stack, indicating a rectifying behavior with a turn-on voltage of about − 2.4 V. This indicates the BTO is n-type semiconducting and forms a Schottky junction with the Pt electrode. Figure 3m insert illustrates the band structure of this heterostructure. In Fig. 3n, here the applied AFM tip bias was set to − 4.3 V and it can be observed that, as the loading force increases from 28nN, to 84nN, to 140nN, and 196nN, the current varies from ~ − 12 nA to − 1 nA. To evaluate the strain distribution of the exfoliated BTO film, particularly under different applied forces 28nN and 196nN, simulation (COMSOL) finite element analysis was performed and the results were shown Fig. 3o, in which the blue region on the film surface represents the AFM tip. This simulation result shows that the gauge factor of flexoelectric BTO with a cubic phase is 1127, which is 4 times larger than that of piezoelectric BTO film with a tetragonal phase. The high gauge factor of the flexoelectric BTO can be attributed to excellent flexoelectric property increased by (1) boosted strain gradient because of de-clamping of BTO from the substrate, different Young’s modulus and Poisson’s ratio of the cubic phase as compared to the tetragonal phase, and the reduced thickness; and (2) high dielectric constant raised by oxygen vacancies in BTO. This research shows that the remote epitaxy of BTO film grown by remote epitaxy has great potential for functional devices in oxide/semiconductor heterogeneous integrated structures [78, 79].

### Optoelectronic application

**Fig. 4:**
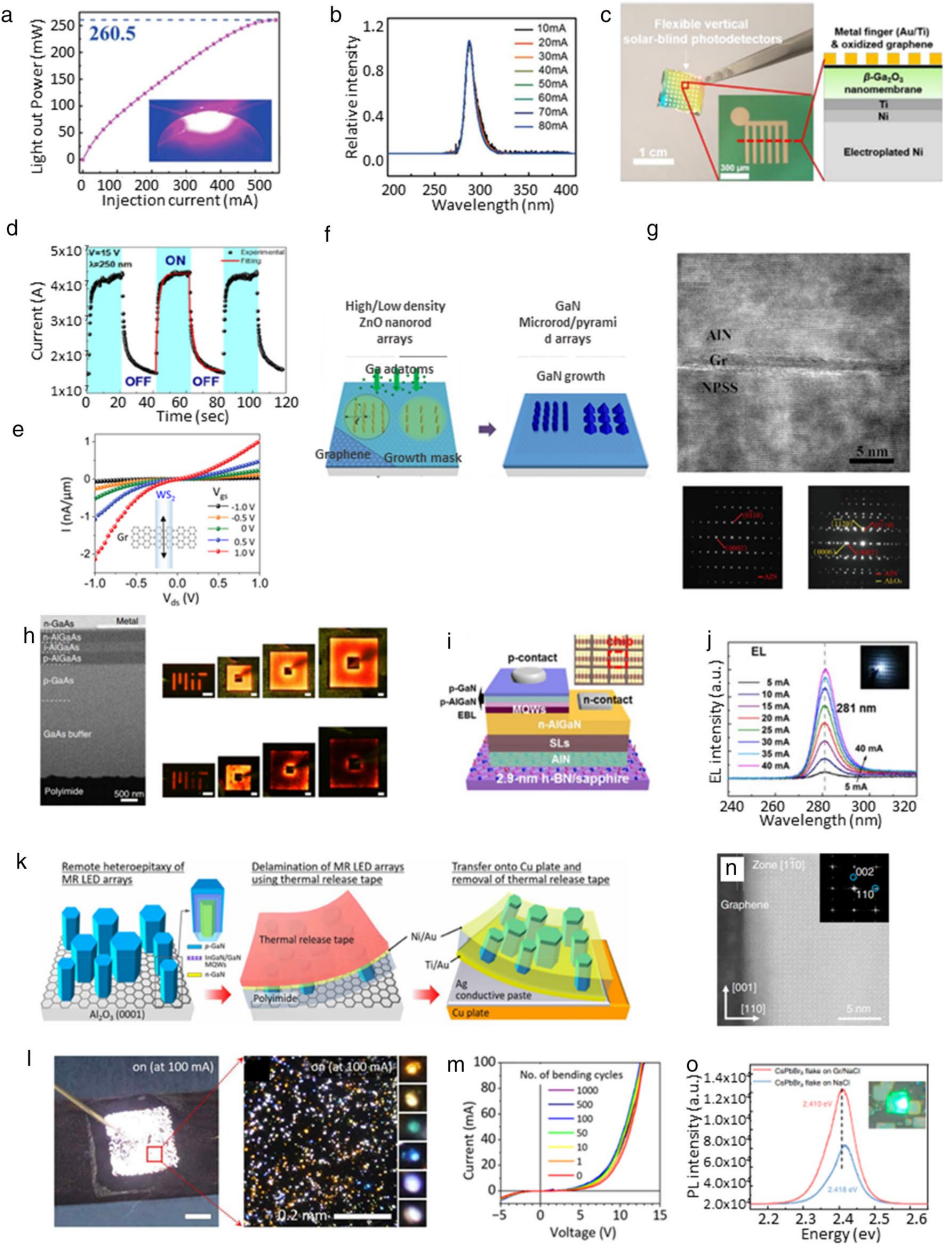
**a**. Light output power curve of violet LED which was grown by the vdW epitaxy [80], **b**. EL spectra of the vdW epitaxial DUV-LED [72], **c**. Optical image of flexible solar-blind photodetectors using β-Ga_2_O_3_ epilayer [34], **d**. UV light on/off cyclic tests of flexible Ga_2_O_3_ photodetector [81], **e**. I-V_ds_ characteristics of WS_2_ epilayer-based photodetector according to V_gs_ [82], **f**. Fabrication procedure of GaN microrod/micropyramid arrays by using the vdW epitaxy [83], g. Cross-sectional high-resolution TEM image and SAED patterns of AlN thin-film grown on the graphene/NPSS [30], h. Cross-sectional SEM image and red light-emitting images of the remote epitaxial AlGaAs LED [90], **i**. Schematic image of DUV-LED on the h-BN/sapphire wafer. The inset image shows a magnified microscopic image of DUV-LED array, **j**. Normalized EL spectra of DUV-LED by various injection currents [91], **k**. Fabrication procedure of the remote epitaxial microrod LED array, **l**. Optical image of microcrystal-based LEDs emitting white light. The right inset displays a magnified photograph of microcrystal-based LEDs, composed of orange, yellow, green, blue, violet, and white-emitting nanocrystals, **m**. I-V characteristic of flexible microrod LED during 1000 bending cycles [101], **n**. Cross-sectional TEM image of the remote epitaxial perovskite thin-film on a polar NaCl wafer. The inset presents FFT patterns of the CPbBr_3_ film, **o**. Steady-state PL curve of the CsPbBr_3_ flakes on the NaCl wafer and the graphene/NaCl substrate. [102]

The III-V/III-N compound semiconductors are representative materials for inorganic thin-film optoelectronic devices including light-emitting diodes (LEDs), solar cells, and photodetectors. Recently, these materials have been spotlighted to realize heterogeneous integration devices since they can be applied to the vdWE and the RE with efficient exfoliation and transfer onto flexible substrates. The vdW growth of a high-quality epilayer could be realized by using selective nucleation of III-N semiconductor materials on the 2D layer. The violet LED was fabricated via the vdW epitaxy since AlN was selectively and directly grown at the optimal nucleation sites [80]. The center of the graphene ring was the best adsorption site, and an AlN composite nucleation layer was formed between the 2D material and GaN epilayer using time-distributed and constant-pressure (TDCP) growth. This method allowed the growth/transfer of high-quality and superior brightness violet LEDs, showing light output power of 260.5 W at 560 mA as shown in Fig. 4a. Deep-ultraviolet (DUV) GaN LEDs were efficiently produced by using quasi-vdW epitaxy with N_2_ plasma-induced AlN nucleation, enabling the 50% reduction of the process speed [72]. The interlayer graphene between the mother wafer and AlN/GaN layers reduced mechanical biaxial stress and the dislocation density in the epilayer due to the weak vdW interaction. The developed DUV-LED exhibited good rectifying characteristic with 4.6 V turn-on voltage and light output power of ~ 1.7 mW with the peak wavelength of 280 nm as shown in Fig. 4b.

Oxide nanomembranes have been also considered as promising materials for achieving 3D hetero-integrated optoelectronic systems. The oxide thin-films were grown by the vdW epitaxy, and transferred to flexible substrates for demonstrating the practical optoelectronic applications [34, 81]. A flexible solar-blind photodetector was realized by using a large-scale β-Ga_2_O_3_ film which was grown by the vdW epitaxy on a compressive-strained epitaxial graphene as shown in Fig. 4c. The graphene grown along the SiC lattice was utilized as a buffer layer of the vdW epitaxy, enabling the successful β-Ga_2_O_3_ nanomembrane growth. The oxide layer was spontaneously delaminated by the Ni stressor-based interface energy modulation, and fabricated to the vertical-structured flexible photodetector. The flexible device displayed excellent photosensitivity of 151.1 A/W at a wavelength of 250 nm due to the wide band gap (~ 4.9 eV) of the β-Ga_2_O_3_ nanomembrane. The vdW epitaxy of β-Ga_2_O_3_ thin-film could be applied to the mica substrate as well as the structure of 2D material/mother substrate because the mica wafer had a stacked structure of 2D-like thin framework layers [81]. The β-Ga_2_O_3_ thin-film grown on the mica was easily exfoliated by mechanical stress from a metal/adhesive tape-structured film, which could be accomplished by intentional destruction of weak vdW bindings between the mica layers. This exfoliation process was universally utilized for Ga_2_O_3_ films regardless of their material phase, film thickness and device-annealing temperature. The exfoliated Ga_2_O_3_ film had an excellent crystallinity due to the high-quality surface property of the mica wafer, presenting self-powered UV detecting performance of 17 mA/W at 0 V bias and 250 nm illumination, as shown in Fig. 4d.

Furthermore, the vdW epitaxy was adapted to form transition-metal dichalcogenides (TMDs) for acquiring high-performance flexible photodetectors. Graphene-WS_2_ (Gr-WS_2_) heterojunction was realized by the nontransfer method of a remote-catalyzed CVD, tungsten oxide deposition, and sulfurization by a plasma-enhanced CVD in successive processes [82]. The developed Gr-WS_2_ heterojunction was conjugated to photodetectors by making Al/AlO_x_ top gate, and Cr/Au-based source/drain contacts. The device was easily transferred to flexible PET film, maintaining its optoelectrical properties of 4.0 A/W photoresponsivity, and fast ON/OFF switching as shown in Fig. 4e.

The vdW epitaxy could be applied not only in 2D-like thin-films but also in micro structured III-N compound semiconductors [20, 21, 30, 34, 83–87]. Figure 4f depicts a schematic procedure of the ZnO nanorod template formation for realizing GaN microrod and pyramid arrays [83]. The ZnO nanorods were selectively grown through the patterned SiO_2_ growth mask on the transferred graphene/SiO_2_/Si substrate, and the GaN LED layers were formed through heteroepitaxy on ZnO in sequence. After metallization and passivation of the microstructure GaN LEDs, heterogeneous integrated devices were fabricated by wet etching the bottom SiO_2_ layer and transferring it onto a heat shrink substrate. The flexible GaN microstructure-based LEDs monolithically emitted blue (wavelength: 505.3 nm) and green light (wavelength: 580.3 nm) according to the microstructures. This multicolor irradiation of the developed LED was attributed to the quantum-confined Stark effect from the different polarity of microrod and micropyramid LEDs. In vdW epitaxy of AlN thin-films on the graphene, nano-patterned sapphire substrate (NPSS) could assist to accelerate the material coalescence, efficiently minimizing the growth time and the process cost [30]. Figure 4g depicts a high-resolution TEM (HRTEM) image and selected area diffraction (SAED) patterns of the AlN film grown on NPSS. The graphene interlayer on NPSS enabled reduction in Al migration barrier, the dislocation density, and the concentrated mechanical strain in the AlN epilayer, enhancing the AlN film quality. Following this research, vdW epitaxial membranes such as compound semiconductors, oxides, TMDs, and microstructured III-N have been applied to various flexible functional optoelectronic devices.

Recently, remote epitaxy has proven significant advantages, including the facile transferability of the vdW epitaxy and the capability to produce freestanding single-crystalline nanomembranes. For instance, III-V thin-films produced from remote epitaxy can be handled by 2DLT to realize diverse photonic devices [7, 42, 88–94] for photonic integrated circuits and exploring nanophotonic physics [95–100]. Figure 4h displays the cross-sectional SEM image and the light-emitting images of flexible AlGaAs LED [90]. Beside the LEDs with visible light (red, green and blue light), DUV AlGaN LED with a 281 nm wavelength was realized by the remote epitaxy, as shown in Fig. 4i [91]. The AlN and DUV LED epilayers were directly grown by the MOPVE method onto the high-quality h-BN layer on the c-plane sapphire wafer. According to TEM analyses, the grown AlN layer had a high crystal quality on 2.9 nm-thick h-BN, showing the wurtzite structure with (0001) c-axis orientation. The flexible tape-attached AlN layer was easily delaminated between h-BN layers owing to the weak adhesion force. The EL peak of DUV LEDs on the AlN/h-BN/sapphire was stably maintained though the increasing injection current from 5 to 40 mA as shown in Fig. 4j.

Remote heteroepitaxy has also been applied to low-dimensional semiconductor materials such as nanowires and microrods for solving the defect issue and the concentrated strain of the epitaxially grown thin-film materials [43, 70]. As displayed in Fig. 4k, III-N compound semiconductor-based microrod LEDs were heterogeneously formed through the graphene layer on the c-plane sapphire substrate [101]. The microrod LED with a p–n junction of core/shell structure was completely passivated with polyimide and transferred to a flexible Cu plate to fabricate a flexible heterogeneous integrated LED. The flexible heterogeneous integrated LEDs maintained their optical and electrical performances with excellent mechanical durability due to the fully-embedded device structure in the polymer passivation layer. Furthermore, as an advantage of the flexible heterogeneous integrated LED, the device was freely cuttable by a normal scissor for attaching onto various versatile curved and conjugated surfaces. Although the flexible device showed white color by the naked eyes, the individual microcrystals emitted different colors of white, orange, yellow, green, blue, and violet in the magnified microscopic images as shown in Fig. 4l. According to EL and PL analyses, various-colored light came from deep-level states in the grown materials, which were closely related with Si doping level and amount of gallium vacancy. Moreover, the heterogeneous integrated LED exhibited excellent opto-electrical properties in a thermal shock test (temperature range from − 40 to 100 ℃) and mechanical fatigue tests with 1000 bending/unbending cycles as shown in Fig. 4m.

The modulation of the dislocation density in a halide perovskite thin-film was theoretically interpreted by using density functional theory (DFT) and molecular-dynamics (MD) simulations, and experimentally confirmed by TEM analyses [102]. Based on the simulations, the electrostatic potential from NaCl and CaF_2_ polar substrate was calculated by the DFT. Figure 4n depicts the STEM image and fast Fourier transform (FFT) pattern of the remote epitaxial CsPbBr_3_ with an orthorhombic lattice structure. The CsPbBr_3_ thin-film showed excellent crystallinity with well-indexed diffraction patterns on the graphene/amorphous NaCl substrate. The kinetic mechanism of the remote epitaxy including thin-film growth, dislocation regulation and strain relaxation was interpreted by MD simulation. The optical property and the effective carrier lifetime were investigated in CsPbBr_3_ films grown by ionic and remote epitaxy. The perovskite film by the remote epitaxy exhibited 3 times higher PL intensity and 4 times longer effective lifetime due to the modulation of the dislocation density as shown in Fig. 4o.

### Electrochemical application

**Fig. 5:**
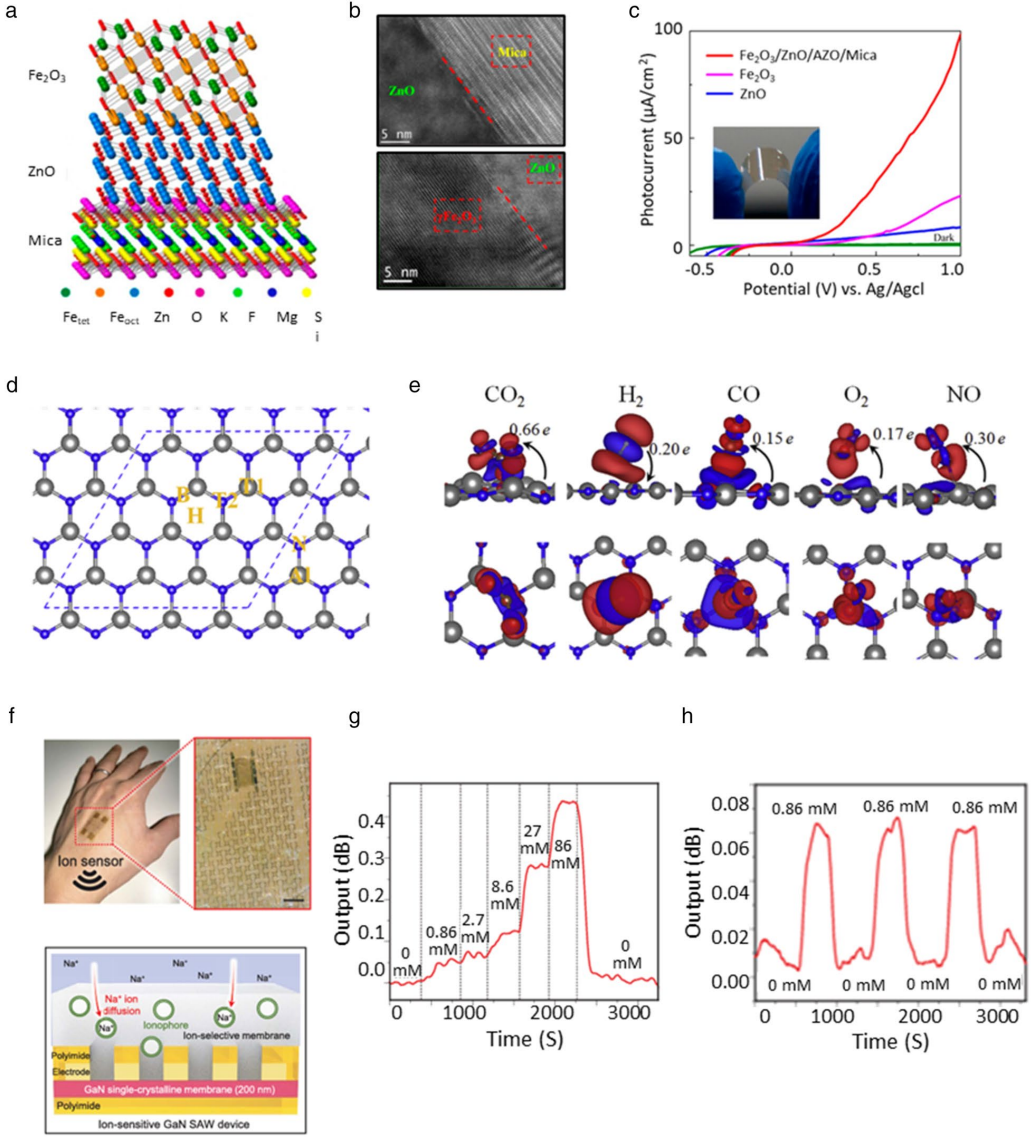
**a**. Structural schematic of the Fe_2_O_3_/ZnO/mica heteroepitaxy, **b**. Cross-sectional TEM image of the ZnO/mica and Fe_2_O_3_/ZnO interface, **c**. Linear-sweep voltammograms of pure Fe_2_O_3_, ZnO, and the Fe_2_O_3_/ZnO electrodes with visible-light illumination and the inset photograph of flexible Fe_2_O_3_/ZnO/mica [114], **d**. The atomic structure of monolayer AlN and four possible adsorption sites, **e**. Charge density difference plots for CO_2_/AlN, H_2_/AlN, CO/AlN, O_2_/AlN, NO/AlN [120], **f**. Photograph of an e-skin attached on the back of hand (top) and Schematic illustrations and wireless ion sensors based on GaN SAW device coated with Na^+^ ion–selective membranes, **g**. Resonant frequency shift in the wireless signals obtained from a GaN SAW ion sensor in response to changes in Na^+^ ion concentration, **h**. Continuous wireless recordings collected from a SAW ion sensor during a series of alternating injections of 0.86 mM NaCl solution and distilled water over the e-skin [74]

For photoelectrochemical (PEC) applications, a heterogeneously integrated structure of necessary materials can simultaneously improve high photocatalytic properties and excellent electron mobility [103–113]. Figure 5a shows the heterostructure of Fe_2_O_3_/ZnO on a mica substrate with high optical transparency and thermal stability [114]. Based on TEM results, high-quality oxide heteroepitaxy of Fe_2_O_3_/ZnO can be fabricated on flexible mica substrate as shown in Fig. 5b. In this structure, the ZnO thin film has high electron mobility, and the Fe_2_O_3_ thin film has chemical stability and efficient light absorption properties in an aqueous environment. Therefore, the high-quality vdW epitaxy PEC (Fe_2_O_3_/ZnO/Mica) structure significantly improves the photocurrent density in the visible light region compared to pure ZnO and Fe_2_O_3_ layers as shown in Fig. 5c. This figure presents the typical linear sweep I–V curves for pure ZnO, Fe_2_O_3_, and Fe_2_O_3_/ZnO electrodes under visible light irradiation (λ > 400 nm). It can be observed that the PEC activity of the Fe_2_O_3_/ZnO heterostructure (98 µA) is much higher than that of pure Fe_2_O_3_ (24 µA) and ZnO (15 µA) films.

For other applications, ultra-thin AlN grown on vdW using MBE equipment has improved electronic properties as well as structural stability, which has expanded the application range [115–119]. The bond between the closest Al and N atoms in AlN is formed by a bond combination of Al-p_z_ and N-p_z_ orbitals. Electrons are transferred from Al to N due to the negative charge difference between Al and N atoms. Therefore, charge transfer from Al to N dominates several properties of ultrathin AlN, including the opening of the band gap. In addition, the Al-N bond of AlN has a dipole moment, which also plays an important role in the adsorption of gas molecules. These features can be useful for sensor applications [93]. Figure 5d shows four different adsorption sites on the AlN surface. Gas molecules are adsorbed on this AlN surface to accept or donate electrons. Figure 5e explains the charge density difference when gas molecules are adsorbed on the surface of AlN [120]. According to the calculation results, H_2_ adsorption causes n-type doping of AlN NSs, while molecules such as CO_2_, CO, O_2_ and NO are acceptors. That is, it makes AlN p-type due to electron charge transfer to gas molecules. This changes the conductivity of AlN, and by measuring the degree of change, gas molecules can be analyzed.

Moreover, SAW sensors can be applied to many fields by using surface acoustic wave deformation [121–134]. Figure 5f shows a heterogeneous integrated structure of ion-selective membrane (ISM) on flexible GaN grown by remote epitaxy [74, 93]. The sensor can monitor the concentration of ions in sweat, which can serve as an indicator for conditions such as hyponatremia, kidney failure and high blood pressure. When specific ions are trapped in the ISM of the heterogeneous integrated sensor, the viscosity and mass of the membrane change, shifting the resonance peak of the SAW sensor, and this movement can be used to detect variation in the ion concentration of the surrounding fluid. Figure 5g, h illustrates the results of in vitro wireless detection of Na^+^ ions using a Na^+^ ISM-coated GaN SAW device on e-skin. Continuous recordings, indicate clear and consistent response and recovery of the output signal, collected by exposing the e-skin to aqueous NaCl solutions containing varying concentrations of Na^+^ ions. The result shows that the detection limit of 0.86 mM represents a small value compared with the biologically relevant range of > 10 mM in sweat, indicating the high sensitivity of the GaN SAW ion sensor.

## Challenge

Remote and vdW epitaxy have several issues in applying 2D template-based growth technology to practical technology despite the extraordinary potential and considerable progress. As the vdW surface does not have a dangling bonds out-of-plane, there is no orbital sharing source during growing materials. Thus, the degree of supersaturation is extremely low compared to the covalent terminated surface, leading to high critical Gibbs free energy. Because of such features, finding optimal growth conditions for single crystalline growth is much more challenging. Of course, in remote epitaxy, remote interaction helps periodic nucleation on vdW surface, but it is still challenging. Also, for the remote epitaxy, it is important to preserve clean interface between 2D materials and substrates and have uniformly thick 2D materials on substrates. There are two methods to form 2D materials on substrates; (i) direct grow on substrates and (ii) transfer on substrates. However, direct growth on substrate is challenging because of difficulty in kinetic control of 2D materials. This causes variation of thickness and random orientation, making it difficult to obtain uniform 2D materials on substrates. In the transfer process, process defects such as wrinkles, holes and residues are always involved. Such defects hinder from having high quality epitaxial layers. They can interfere with remote interaction, disrupting atomic guidance. Also, defective sites generate covalent bonds at the interface between grown materials and substrates, blocking the 2DLT process. Therefore, to overcome these limitations, a thorough investigation of all kinetic parameters that affect the growth process is necessary. Moreover, the transfer process conditions that minimize stress on 2D materials need to be optimized. Through these efforts, the limitations can be overcome, and it is expected that these solutions will lead to further innovations in the future.

## Opportunity

Silicon is an abundant element on earth and dominantly used in various fields including integrated circuits, sensors, and solar cells. However, as technology advances and the required specifications on the devices are increasing, the necessity of materials with high performance is increasing. These things can be overcome by compound materials such as GaN, GaAs, InP, etc. outperform silicon, but it is difficult to use the material due to the high production cost. This problem can be solved by layer transfer technology, and advanced growth method of remote and vdW epitaxy also suggest a new type of layer transfer technology, 2DLT. The 2DLT transfer technology together with these growth methods can rapidly release high-quality growth materials and reuse expensive substrates, effectively reducing production costs. This provides an opportunity to mass-produce high-performance devices essential in a ubiquitous environment at low cost. Moreover, these technologies enable stacking multiple freestanding materials on a single host substrate. The possibility of integrating heterogeneous materials allows for implementation of multifunctional devices that could not be implemented in one device. In addition, new heterogeneous structures provide a platform to discover new physical coupling phenomena that have not been realized. We believe that these opportunities could provide a solution to current technical challenges as well as a guide to innovative technology in the future.

## Data Availability

The review is based on the published data and sources of data upon which conclusions have been drawn can be found in the reference list.
